# #Acne: A Thematic Qualitative Analysis of Acne Content on TikTok

**DOI:** 10.1111/ajd.14433

**Published:** 2025-02-26

**Authors:** Laxmi Iyengar, Susan Saldanha, Alvin H. Chong

**Affiliations:** ^1^ Skin Health Institute Carlton Victoria Australia; ^2^ Department of General Practice Monash University Melbourne Victoria Australia; ^3^ Department of Medicine (Dermatology) St. Vincent's Hospital Melbourne, the University of Melbourne Melbourne Victoria Australia

**Keywords:** acne, medical education, qualitative research, TikTok

## Abstract

**Aim:**

TikTok has accrued over 3 million posts and 129 billion views under #acne, establishing itself as a popular platform amongst adolescents to access health information. We conducted an in‐depth thematic analysis of acne videos on TikTok to determine how adolescents engage with acne content.

**Methods:**

The top 150 consecutive videos under #acne on TikTok were subjected to rigorous qualitative analysis by experienced researchers (Laxmi Iyengar, Susan Saldanha) until data saturation was reached, guided by SRQR (Standards for Reporting Qualitative Research) guidelines. Five themes were identified:(1) Pimple popping, (2) Acne education, (3) ‘Acne transformation’ depicting treatment success stories, (4) Acne positivity, normalising acne and (5) Acne reality, capturing the lived experience of acne.

**Results:**

Overall, the top 150 videos under #acne accumulated almost 2 billion views and 102 million followers. The majority of acne content was uploaded by females (125/150; 84%). Medically trained clinicians represented only 11% of the content (17/150). Pimple‐popping videos attracted the most significant viewership (804 million views; 44%; 17% of content) despite having a lower proportion of content than acne education videos, uploaded by patients and clinicians (324 million views; 34% of content). These videos included dietary hacks, miracle cures and narratives that antagonised the medical treatments. Acne reality videos demonstrating the mental health burden of acne were the least popular amongst TikTok viewers (79 million views; 4% of content).

**Conclusion:**

Thematic qualitative research of acne on TikTok bridges the gap between clinical expertise and the lived experiences of those navigating acne in the digital age. Based on the results of the study, strategies are proposed for dermatologists to engage in the TikTok platform on the topic of acne, including medicolegal precautions. Given the lack of content credibility on social media, this research urges dermatologists to redefine healthy skin care practices based on evidence‐based principles.

## 1 Introduction

TikTok has emerged as one of the fastest‐growing social media platforms with over 1.04 billion monthly active users worldwide [1]. Known primarily for its entertainment and communication features, TikTok has transcended as the most popular platform through which adolescents access a wide range of information [2], including acne‐related content.

Adolescents, often referred to as Gen Z, represent the vast majority of TikTok users (44.7%) [2]. Compared to other social media platforms such as WhatsApp, Facebook, YouTube or Instagram, TikTok features a distinctive interface that encourages instantaneous content consumption and creation, fostering a sense of spontaneity and creativity that aligns well with an adolescent mindset [3]. To date there are 3 million individual posts under #acne. This is not surprising given that acne is a common condition afflicting 9.4% of the global population [4]. Acne can impact self‐esteem during adolescence, as sufferers often face stigma and discrimination. Depression and psychological disorders are also prevalent in patients with acne and significantly impact the self‐worth of individuals [5, 6].

There is also a plethora of medical and non‐medical treatments for acne in the community, creating further confusion and angst amongst youth [7]. Many of these treatments are expensive, potentially harmful and lack scientific evidence, and yet can be endorsed by advertising and sponsorship [8, 9]. Interestingly, a number of medically validated acne treatments (e.g., isotretinoin) are also associated with an array of side effects, and changes to mood are reported [10, 11]. Overall, this has resulted in a conflicted landscape urging those afflicted with acne to do their own research, seek information, and listen and learn from lived experiences. Information accessed via social media platforms therefore presents an attractive avenue to achieve this goal.

Whilst the ease of information access and sense of community are advantages of social media platforms such as TikTok, the rapid spread of misinformation is also of concern [12, 13]. TikTok users gather information, share personal stories and “hacks”, disseminate information on acne treatment, and build communities of online users who actively engage with this information. Many of these users are recognised as ‘skinfluencers’, whose views and impressions have a tremendous impact on young users who follow emerging trends [14]. ‘Skinfluencers’ are often sought after by marketing giants to promote a product, further resulting in the spread of misinformation in the community.

There is no accountability for the validity of information posted on TikTok, and there is a paucity of evidence‐based dermatologic information [12, 13]. Medical professionals disseminating health information represent a small minority of TikTok users [14]. Users posting and sharing information are enticed by the number of views per video, aiming to go viral and reach overnight celebrity status [14, 15]. The ability to ‘tag’ and share messages amongst diverse communities, ‘like’ videos and comment allows information to be disseminated at an exponential rate to billions of Upon joining the app, users are initially presented with general content. Over time, the platform customises the content based on the types of reels viewed, the duration spent watching specific videos, and user interactions such as likes, comments, and follows. The content typically consists of short videos ranging from 5 s to 10 min, often featuring a single individual. Users can engage by liking, following, and commenting on the content, while creators can respond directly, fostering a highly interactive experience. By posting and accessing information on acne, TikTok algorithms continue to feed the vulnerable user with further personalised curated information on the topic and advertising material [14, 15]. Critics argue that TikTok therefore enhances body stereotypes and body humiliation trends which can be detrimental to young people [14].

In contrast, emerging trends have also demonstrated that given its reach, TikTok can be used to promote body positivity and neutrality and redefine body image and self‐worth [16]. It is also a powerful platform for medical engagement and to advance public health awareness and literacy [16, 17]. A small number of health professionals have also started to use TikTok for medical education, raising awareness for skin health and counteracting the negative influence of propaganda and misinformation [17, 18].

In the present study we conduct an in‐depth qualitative analysis to understand how acne content is portrayed on TikTok and identify how young people engage with acne content. This information is critical to enable medical professionals to understand how adolescents engage with acne content on TikTok and provide answers to the questions that patients are genuinely seeking, deliver relevant medical education and optimise treatment outcomes. We address the medicolegal issues clinicians may face posting on TikTok and propose strategies for the dermatologist to engage and educate using this platform, whilst upholding the principles of clinical professionalism.

## 2 Methods

**FIGURE 1:**
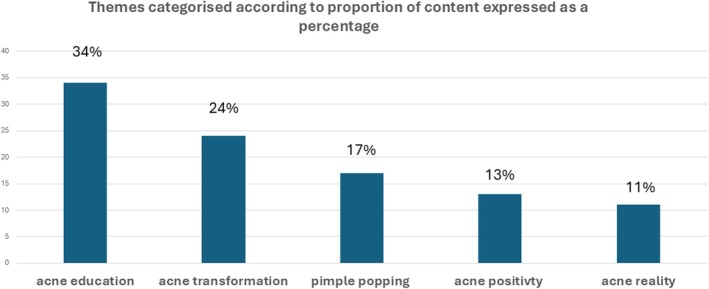
Themes categorised according to the proportion of content on TikTok.

This project was approved by the Monash Human Research Ethics Committee (ID:38935). A total of 150 consecutive videos under #acne were extracted on the 25th September 2023. Two independent researchers (Laxmi Iyengar, Susan Saldanha) with previous experience in qualitative research were involved in critically reviewing video content and thematic analysis, guided by SRQR guidelines (Table S1). Alvin H. Chong is a consultant dermatologist who provided clinical oversight and supervision for the project. Overall, there was a high degree of intercoder reliability between the researchers (Figure 1).

Descriptive quantitative analysis recorded included categorical data such as the length of video, the number of views per video, the number of comments, likes and shares to determine the popularity of the video. Qualitative analysis of acne content on TikTok was conducted according to previously published protocols [19, 20]. For thematic analysis, LI and SS developed themes by watching 50 videos to initially derive themes and a codebook through an inductive approach. A total of 150 videos were then independently coded by each researcher until data saturation had been reached, referring to the point at which no further themes or insights emerged from further data collection. If themes overlapped, the major theme was identified based on comments evoked by viewers in response to the video. User anonymity was maintained by assigning pseudonyms, and all comments and demographic information were de‐identified. While consent was not directly obtained from each TikTok presenter, consent was implied as TikTok users are aware that any public activities on the platform may be read, collected or used by third‐party users as outlined in their privacy policy [21].

## 3 Results

Overall, the top 150 videos under #acne accounted for over 2 billion views and over 102 million followers (Table 1). The majority of acne content on TikTok was uploaded by females (125/150; 84%). Medically trained clinicians contributed to only 11% of the acne content uploaded on TikTok (17/150). Video durations ranged between 5 s and 5 m 17 s, with an average duration of approximately 33 s. Data analysis revealed that pimple‐popping videos were the most popular, accumulating 804.4 million views, 176.1 K comments, and 87.7 K shares. Approximately 14% of videos (21/150 videos) directly endorsed a product, including both topical treatments endorsed by skinfluencers as well as patients who had successfully treated acne with isotretinoin.

**TABLE 1:** Content analysis of 150 TikTok videos.

Content analysis of 150 TikTok videos	Content
Gender distribution	25/150 (male); 125/150 (female)
Videos by board certified clinicians	17/150 (11%)
Videos that endorsed a product	21/150 videos
Longest video	5 m 17 s; acne education
Video with the most number of shares	87.7 K; pimple popping
Person with the most number of followers	18.2 M; acne education
Video with the most number of comments	176.1 K; pimple popping
Video with the most number of total views	201.1 M; pimple popping

Five major themes were identified from the analysis: (1) pimple popping, (2) acne education, (3) acne treatment success stories termed as ‘acne transformation’, (4) videos on acne positivity that normalised acne as beautiful, and (5) acne reality that captured the lived experience of acne (Table 2, Figure 1).

**TABLE 2:** Themes categorised according to the proportion of content of 150 TikTok videos.

Theme	Proportion of content
Acne education	51/150 (34%)
Acne transformation	36/150 (24%)
Pimple popping	26/150 (17%)
Acne positivity	20/150 (13%)
Acne reality	17/150 (11%)

### 3.1 Theme 1: Pimple‐Popping

Pimple‐popping videos were typically lengthy (> 1 min) and set to trending music, focused on comedone/cyst extraction with minimal engagement from the person posting the videos. These videos were typically posted by aestheticians, who endorsed these procedures, with accompanying statements such as:

relax with me [22]

or

so satisfying [23]

**FIGURE 2:**
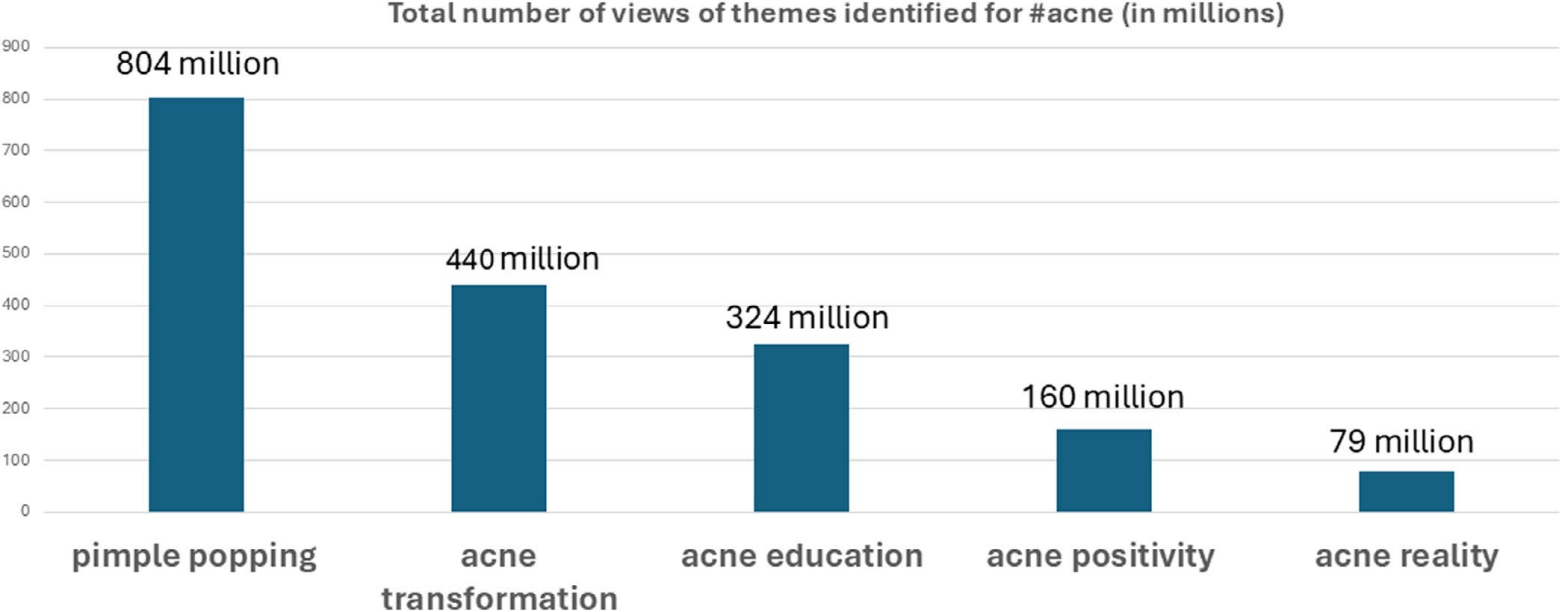
Popularity of acne content on TikTok.

Despite the lack of interaction, pimple‐popping videos were by far the most popular, receiving the majority of total views (804.4 million) during the study period, although they only represented 17% (26/150) of the total content under #acne (Table 3, Figure 2).

**TABLE 3:** Themes categorised according to number of views.

Theme	Total number of views
Pimple popping	804 million (44%)
Acne transformation	440 million (25%)
Acne education	324 million (17%)
Acne positivity	160 million (10%)
Acne reality	79 million (4%)

### 3.2 Theme 2: Acne Education

Acne education represented 34% (51/150) of content under #acne and accrued 324 million views. Videos within this theme were created by both patients and clinicians. Much non‐clinician‐led educational content often included dietary hacks such as the use of apple cider vinegar, personal success stories such as the use of salt on skin, or alternative treatments that antagonised medically endorsed treatments and promoted misinformation about the cause of acne. Education regarding the causes of acne included persuasive commentaries such as:

I love me some good skin care, but that's not enough when it comes to your skin. You need to start from the inside out. Prioritise gut health and start balancing your hormones. A tonne of food can be huge acne triggers. [24]

Medically trained clinicians created 17/150 videos, representing only 11% of the total content under #acne, targeting acne education. It was unclear whether this content received brand sponsorship, as there were no formal disclosures associated with the videos. Many acne education videos were posted by the same individual, suggesting some may have been skinfluencers.

Clinician‐led tutorials for acne management were supplemented with instructive statements such as:

Acne treatment can seem overwhelming, but here is a basic primer. [25]

### 3.3 Theme 3: Acne Transformation

Acne transformation videos, showcasing ‘glow‐ups’, and success stories with isotretinoin or topical treatment represented 24% (36/150) of the content of TikTok and received 440 million views. The sponsorship status of these videos was often ambiguous, although many videos were accompanied by endorsements from brands who supported the videos with statements such as:

Thanks for being a cicaplast lover. [26]

In addition, the comments associated with acne transformation videos positively reinforced the improvement of acne. These videos generally depicted a historical timeline, outlining the humiliation and trauma initially associated with the acne journey and the glory and triumph associated with the resolution of acne in response to specific treatments:

It's so weird looking back at how far my skin has come; genuinely never thought I'd have clear skin. Sending my love to anyone struggling with acne; it gets better. [27]

Being vulnerable, showing my biggest insecurity, but finally seeing results after 1month of being on Roaccutane. [28]

They say there are five stages of grief: denial, anger, bargaining, depression, acceptance—REVENGE. [29]

### 3.4 Theme 4: Acne Positivity

Videos on acne positivity promoted body acceptance and normalised acne as beautiful and even attractive for TikTok users. TikTok users with acne were portrayed as martyrs and demonstrated the bravery associated with their acne journey. Many of these videos focused on building an acne community, encouraging engagement with the viewer and utilising comments and banners to engage the viewer. Videos that promoted acne positivity represented 160 million total views, and 13% (19/150) of the content on TikTok under #acne.

There is no shame in having acne; it doesn't change a single thing about you.

This morning I couldn't help but cry; my acne is giving me so much pain, it's so inflamed, it's hard to wash my face without it hurting. But it's not always going to stay like this, and I've got to stay positive. It's okay to have bad days, but you've got to pick yourself up.

My messages are always open if anyone wants to talk. [30]

Such personal and emotive remarks invoked viewers to reciprocate and engage in conversation related to acne positivity, translating to the normalisation of acne.

### 3.5 Theme 5: Acne Reality

Acne reality videos demonstrated the mental health impact of acne and the lived experience of those affected. Acne reality videos were the least popular of the five themes identified, evidenced by the smallest number of views (79 million in total) and also representing the least amount of content under #acne (17/150; 11%).

The mental health impact of acne demonstrated in videos was poignantly illustrated by quotes linked to the videos, such as:

Acne isn't just pimples.

It's being scared to go out.

It's feeling the urge to look at yourself in the mirror to see if you have new pimples.

It's always comparing your skin to others.

It's being scared to eat things that could trigger your acne.

It's feeling powerless.

It's feeling the need to wear makeup wherever you go.

It's thinking that everyone thinks you're ugly.

It's never being satisfied with how your skin looks like, even if it gets better. [31]

Many patients expressed frustration with the array of acne treatments promised to be miracle cures for acne, captured elegantly in the sentiments expressed in the following quotation:

It's a different cry when you are tired and frustrated of trying different expensive products for acne and making it worse. And it's the first thing people notice about you. [32]

The lived experience of living with acne, including the stigma and discrimination experienced by patients with acne, was captured below:

Turning 24 this year with declined opportunities and discouraged dreams because of my acne marks and breakouts. [32]

## 4 Discussion

This study delves into the intricate landscape of acne‐related content on TikTok through a qualitative analysis of 150 videos under the #acne hashtag. The in‐depth thematic exploration reveals how deeply young people engage with and are influenced by this content. These findings illuminate critical opportunities for healthcare professionals to step back and truly understand the patient's perspective, offering a pathway to bridge the gap between clinical expertise and the lived experiences of those navigating acne in the digital age.

Pimple‐popping videos and acne transformation stories were by far the most popular content. The popularity and fascination with pimple popping is consistent with earlier studies [33]. fMRI studies of the brain have shown specific patterns of brain activity in the nucleus accumbens and insula (coding of aversion/pleasure) and the frontopolar region (prediction of outcomes of motor decisions) associated with the pleasure and aversion linked to watching these videos [33]. Similarly, the popularity of watching acne transformation success stories is supported by neuropsychological studies [34]. Zak has proposed that emotionally engaging narratives cause oxytocin release and inspire post‐narrative actions by virtue of altering behaviour and attitudes [34]. The popularity of acne treatment success stories, therefore, is not surprising and is supported by ‘gratification theory’, reinforced by feeds curated by the TikTok algorithm based on previous views, trending music and a sense of camaraderie with a virtual acne community [35, 36].

This research highlights both the positive and negative impacts of TikTok. On the positive side, the platform increases awareness about isotretinoin, promotes acne normalisation, and provides users with opportunities to engage with content creators about their personal acne journeys. However, it also facilitates the rapid spread of misinformation about certain products, including medically validated ones, and elevates non‐expert influencers to celebrity status. Furthermore, the findings reveal that many adolescents with acne appear to lack awareness of available treatments, face barriers to accessing them, or struggle to find accurate information on acne management. This underscores the need for clinicians and dermatologists to actively engage on TikTok to provide reliable, evidence‐based guidance. Interestingly, the majority of the content reviewed under #acne encompassed acne education (34%). This suggests that TikTok users are actively seeking answers to questions that clinicians may have overlooked, urging dermatologists to make a positive contribution to this space. Extensive research has shown that the quality of dermatology education on TikTok varies widely, with much of the patient‐led education based on anecdotal evidence [37]. The vulnerability of adolescent subscribers in the social media space is therefore of particular concern, given that many such treatments can cause harm, perpetuated by the negative impact of acne itself on an individual's mental health [38] As a corollary to this, videos that promoted acne positivity shed light on ways in which social media can be used to build positive connections and social capital [39], thereby promoting acceptance, redefining biological norms and counteracting the unrealistic beauty goals of ‘glass skin’, united against the dominant rhetoric.

The demographic patterns observed in the analysed videos align with previous studies [14, 40]. Zheng and colleagues [14] previously demonstrated that the majority of acne‐related content is uploaded by females and that medically trained professionals represent a small minority of users. This results in a predominance of low‐quality educational content from a medical standpoint, a concern echoed by this study. Of note, a recent cross‐sectional analysis of content on TikTok showed that videos from medical and non‐medical professionals received similar levels of engagement [41]. This has raised concern amongst many healthcare professionals and has sparked interest for dermatologists to supersede the social media space to counteract the propaganda and misinformation, enabling opportunities for TikTok users to access reliable, evidence‐based information [17]. The major barriers that have been cited to explore clinician under‐representation include time, lack of positive outcomes, harassment, lack of social media training and support [42].

Navigating medicolegal obligations can be challenging for clinicians engaging on social media platforms. Principles of medical professionalism must be upheld, such as maintaining patient confidentiality, promoting evidence‐based health information, maintaining collegiality, ensuring cultural safety and avoiding personal advice or clinical care through these platforms. They should be mindful that shared content is public and can be misinterpreted [42, 43, 44, 45]. To mitigate medicolegal risks, clinicians should review posts for any perceived violations of patient dignity, avoid reinforcing stereotypes, and abstain from cyberbullying [42, 43, 44, 45]. Patient consent for social media content, using disclaimers, disclosing conflicts of interest, and respecting copyright laws are key practices to protect both clinicians and patients [42, 43, 44, 45].

### 4.1 Limitations

In the present study we analysed the top 150 videos in consecutive order based on the search term #acne. This generally represented the most popular videos; therefore, content with fewer views or engagement might offer additional insights into themes that may be unexplored. In addition, while the comments included in the thematic analysis enrich the trends observed, it is essential that these accurately represent the vision of the content creator. All such analysis is necessarily subjective, influenced by a range of emotions evoked and personal experiences. Although two researchers independently coded themes, the interpretations of the researcher may have inadvertently impacted the data presented.

## 5 Conclusion

This study provides insight into how young people interact with acne‐related content on TikTok, revealing opportunities for presenting evidence‐based dermatology information to this audience. While the risks associated with social media are widely recognised, this research emphasises the importance of understanding specific information that young people are actively seeking. By tapping into these insights, dermatologists are uniquely positioned to lead the charge in redefining healthy skincare practices and behaviours, ultimately leading to improved clinical practice by fostering an informed and supportive landscape for patients.

As well as gaining a genuine understanding of the patient perspective, by increased representation on social media platforms, dermatologists can clarify misconceptions and provide factual, evidence‐based answers to questions that have been overlooked in clinical practice in a patient‐centred framework that resonates with young people. Dermatologists can raise awareness about healthy skin care practices, provide access to research‐driven written resources and links to medical and emotional support services, and counteract misinformation and the negative stereotypes perpetuated by social media.

This research also suggests specific strategies for dermatologists to educate about acne on TikTok. As viewer attention spans are potentially short, video content needs to be succinct (ideally less than 30 s) and engaging, with an impactful opening (e.g., a pimple being popped, trending music, or eye‐catching banners). Videos may be augmented with images, and the personality of the content provider should shine through, exuding energy and vibrance to connect with a younger audience. Videos should avoid excessive medical jargon and be skilfully crafted using language and trends that resonate with Gen Z.

In addition, the personal experience shared by patients is highly valued, with personal testimonies representing the majority of dermatologic content (47%) [46]. With patient consent, dermatologists could consider collaborating with their acne patients to present meaningful and impactful content on TikTok. Furthermore, to improve engagement, content needs to be regularly created and updated as videos are viewed in succession based on TikTok's algorithm, which recommends content based on prior engagement (time watched, comments, likes, tags, shares), and followers also expect new content for infotainment.

Medical education on social media led by dermatologists is not just an opportunity; it is a call to action to transform the way acne care is approached in the digital age.

## Author Contributions

L.I. and S.S. are two independent researchers that conducted this qualitative study. L.I. researched the project and wrote the manuscript. A.H.C. provided clinical oversight and supervision and reviewed the manuscript.

## Ethics Statement

This project was approved by the Monash Human Research Ethics Committee (ID: 38935).

## Conflicts of Interest

The authors declare no conflicts of interest.

## Supporting information


Table S1.

## Data Availability

The data that support the findings of this study are openly available at https://pubmed.ncbi.nlm.nih.gov/ and https://www.tiktok.com.
