# Supplementary material for: #Acne: A Thematic Qualitative Analysis of Acne Content on TikTok
Source: Australas J Dermatol. 2025 Feb 26;66(3):127–34. doi: 10.1111/ajd.14433 (PMC12062723; doi:10.1111/ajd.14433)
Supplement: Supplementary file 1 — Table S1. [file AJD-66-127-s001.docx]

SUPPLEMENTARY MATERIAL

**Codebook used for thematic qualitative analysis of acne content on TikTok**

| **Theme** | **Subthemes** | **Code Description** | **Key references** |
| --- | --- | --- | --- |
| 1.Pimple popping | n/a | Defined as videos where pimples/cysts/comedones/sebaceous filaments are extracted without any further information or engagement | 22,23 |
| 2.Acne education | 2a. Clinician-driven acne education and management  2b. Patient driven information regarding causes of acne  2c. Misinformation and theories about causes of acne  2d. Alternative treatments, dietary fads  2e. What is the expertise/background of the person posting the video?  2f. What is the level of evidence? Is the information based on anecdotal experience? | Defined as clinician led or patient led videos providing suggestions to improve acne or the underlying cause of acne | 24,25 |
| 3.Acne transformation | 3a. Isotretinoin-driven acne treatment success stories  3b. Topical treatment driven acne treatment success stories  3c. self-esteem/confidence, bravery associated with acne journey | Defined as videos showing a dramatic improvement of acne with the use of topical  products or isotretinoin (Roaccutane) | 26,27,28,29 |
| 4. Acne positivity | 4a. Acne is normal and beautiful  4b. Acne relatability  4c. Celebrity acne journey stories  4d. Acne community | Defined as videos that normalise  acne to the broader community | 30 |
| 5. Acne reality | 5a. Mental health impact of acne.  5b. Experience of being bullied or feeling discriminated due to acne | Defined as videos showing the  lived experience of those suffering  from acne, including the  mental health impact, frustration and stigma associated with acne | 31,32 |
